# Identification of novel fusion genes in lung cancer using breakpoint assembly of transcriptome sequencing data

**DOI:** 10.1186/s13059-014-0558-0

**Published:** 2015-01-05

**Authors:** Lynnette Fernandez-Cuesta, Ruping Sun, Roopika Menon, Julie George, Susanne Lorenz, Leonardo A Meza-Zepeda, Martin Peifer, Dennis Plenker, Johannes M Heuckmann, Frauke Leenders, Thomas Zander, Ilona Dahmen, Mirjam Koker, Jakob Schöttle, Roland T Ullrich, Janine Altmüller, Christian Becker, Peter Nürnberg, Henrik Seidel, Diana Böhm, Friederike Göke, Sascha Ansén, Prudence A Russell, Gavin M Wright, Zoe Wainer, Benjamin Solomon, Iver Petersen, Joachim H Clement, Jörg Sänger, Odd-Terje Brustugun, Åslaug Helland, Steinar Solberg, Marius Lund-Iversen, Reinhard Buettner, Jürgen Wolf, Elisabeth Brambilla, Martin Vingron, Sven Perner, Stefan A Haas, Roman K Thomas

**Affiliations:** Department of Translational Genomics, Center of Integrated Oncology Cologne–Bonn, Medical Faculty, University of Cologne, 50924 Cologne, Germany; Genetic Cancer Susceptibility Group, Section of Genetics, International Agency for Research on Cancer (IARC-WHO), 69008 Lyons, France; Computational Molecular Biology Group, Max Planck Institute for Molecular Genetics, D-14195 Berlin, Germany; Department of Systems Biology, Columbia University, New York, NY 10032 USA; Department of Prostate Cancer Research, Institute of Pathology, Center for Integrated Oncology Cologne-Bonn, University Hospital of Bonn, Bonn, Germany; Blackfield AG, Gottfried-Hagen-Str. 60, 51105 Cologne, Germany; Department of Tumor Biology and Genomics Core Facility, Norwegian Radium Hospital, Oslo University Hospital, N-0310 Oslo, Norway; Center for Molecular Medicine Cologne (CMMC), University of Cologne, 50931 Cologne, Germany; Department I of Internal Medicine, Center of Integrated Oncology Cologne-Bonn, University of Cologne, 50924 Cologne, Germany; Network Genomic Medicine, University Hospital Cologne, Center of Integrated Oncology Cologne-Bonn, 50924 Cologne, Germany; Max Planck Institute for Neurological Research, 50931 Cologne, Germany; Cologne Center for Genomics (CCG), University of Cologne, Cologne, 50931 Germany; Cologne Excellence Cluster on Cellular Stress Responses in Aging-Associated Diseases (CECAD), University of Cologne, Cologne, Germany; Institute of Human Genetics, University of Cologne, 50931 Cologne, Germany; Bayer Schering, Berlin, Germany; Department of Pathology, St. Vincent’s Hospital, Melbourne, 3065 Victoria Australia; University of Melbourne Department of Surgery, St Vincent’s Hospital, Melbourne, 3065 Victoria Australia; Department of Haematology and Medical Oncology, Peter MacCallum Cancer Centre, Melbourne, 3002 Victoria Australia; Institute of Pathology, Jena University Hospital, Friedrich-Schiller-University, 07743 Jena, Germany; Department of Internal Medicine II, Jena University Hospital, Friedrich-Schiller-University, 07743 Jena, Germany; Institute for Pathology Bad Berka, 99438 Bad Berka, Germany; Institute of Clinical Medicine, Faculty of Medicine, University of Oslo, N-0424 Oslo, Norway; Department of Oncology, Norwegian Radium Hospital, Oslo University Hospital, N-0310 Oslo, Norway; Department of Thoracic Surgery, Rikshospitalet, Oslo University Hospital, N-0027 Oslo, Norway; Department of Pathology, Norwegian Radium Hospital, Oslo University Hospital, N-0310 Oslo, Norway; Department of Pathology, University Hospital Medical Center, University of Cologne, 50937 Cologne, Germany; Department of Pathology, CHU Grenoble INSERM U823, Institute Albert Bonniot, 38043 CS10217 Grenoble, France

## Abstract

Genomic translocation events frequently underlie cancer development through generation of gene fusions with oncogenic properties. Identification of such fusion transcripts by transcriptome sequencing might help to discover new potential therapeutic targets. We developed TRUP (Tumor-specimen suited RNA-seq Unified Pipeline) (https://github.com/ruping/TRUP), a computational approach that combines split-read and read-pair analysis with *de novo* assembly for the identification of chimeric transcripts in cancer specimens. We apply TRUP to RNA-seq data of different tumor types, and find it to be more sensitive than alternative tools in detecting chimeric transcripts, such as secondary rearrangements in *EML4-ALK*-positive lung tumors, or recurrent inactivating rearrangements affecting *RASSF8*.

**Electronic supplementary material:**

The online version of this article (doi:10.1186/s13059-014-0558-0) contains supplementary material, which is available to authorized users.

## Background

Genomic rearrangements in cancer often lead to gene fusions disrupting the activity of tumor suppressor genes or activating proto-oncogenes, thus playing an important role in tumor development. Gene fusions can lead to the constitutive activation of a kinase, on which cancer cells become dependent, a process sometimes referred to as ‘oncogene addiction’ [1]. One of the big successes in the treatment of cancer was the identification of small molecules that specifically target fusion proteins, such as imatinib for CML patients carrying the *BCR*-*ABL* translocation [2] or crizotinib in the case of *EML4-ALK* positive lung tumors [3].

Paired-end transcriptome sequencing (PE RNA-seq) is a powerful tool for the identification of fusion transcripts in tumors [4]. However, the complexity of the cancer transcriptome, the high dynamic range of gene expression, and the prevalence of sequencing errors confound the computational fusion detection from RNA-seq data [5]. Existing methods in this field primarily rely on read-pair analysis by assuming that deviations of the mapping distance or orientation are caused by fusion events [6,7]. To increase sensitivity, a split-read mapping method may be adopted in addition to read-pair analysis [8,9]. However, the short reads typically generate a large number of candidates including many false positives that need sophisticated further processing, which is computationally expensive. It has recently been shown that *de novo* assembly of novel junctions in a targeted region obtained by read-pair analysis leads to accurate fusion predictions, since it provides high quality and longer sequences spanning the fusion point by leveraging dependency among short reads [10].

In this study we present TRUP, a computational pipeline that combines split-read and read-pair analysis with *de novo* assembly of candidate regions containing a potential breakpoint, to achieve sensitive and accurate detection of fusion transcripts. TRUP afforded detecting secondary in-frame rearrangements in *EML4-ALK*-positive lung adenocarcinomas, as well as the identification of recurrent inactivating rearrangements affecting the candidate tumor suppressor gene *RASSF8*.

## Results and discussion

### TRUP: A pipeline for detecting fusion genes in cancer

**Figure 1:**
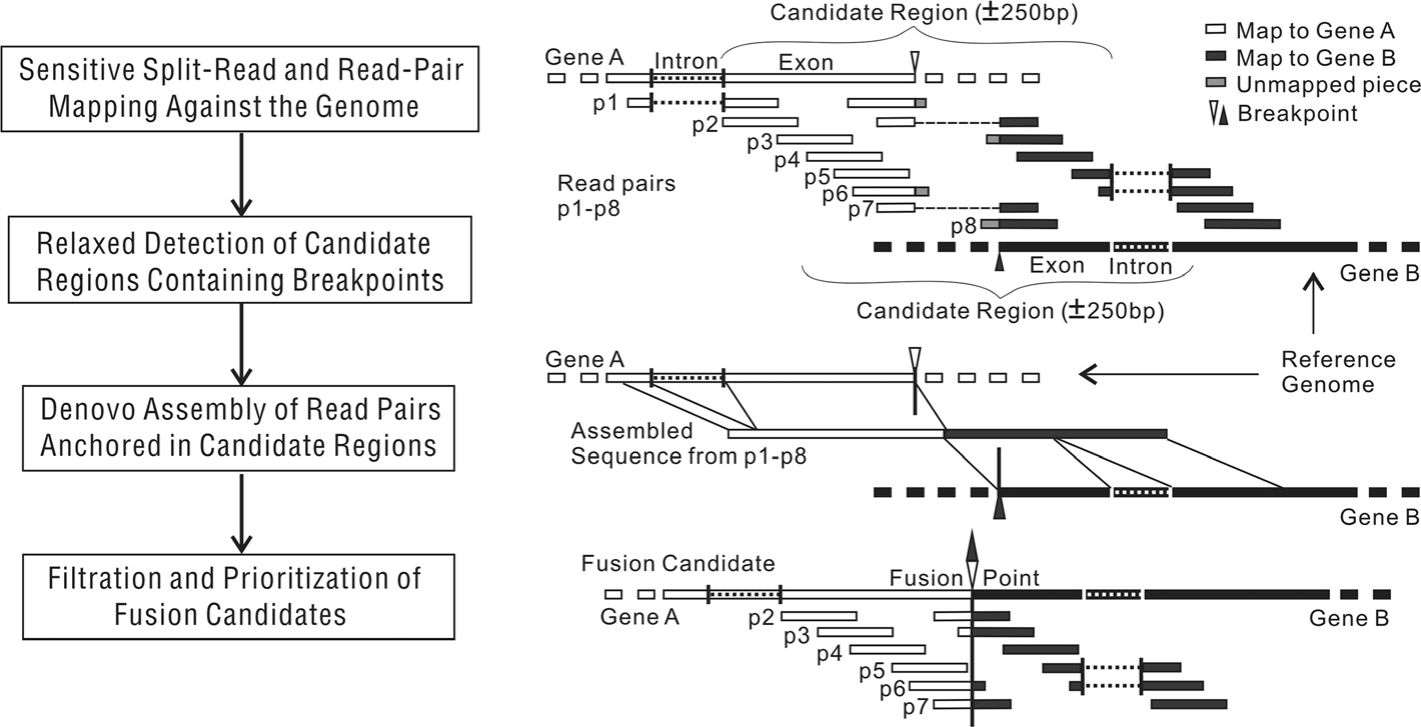
**Overview of the TRUP pipeline.** The schematic diagram on the left panel shows the four major processing steps applied in TRUP. The cartoon on the right panel illustrates an example of detecting a fusion event. White and black colored boxes indicate reads mapped to gene A and to gene B, respectively. In a first step, TRUP aligns the read pairs onto the genome allowing discovery of chimeric alignments (read pair id p2 and p7 in the cartoon) and partial alignments (p1, p3, p6, and p8). To guarantee a sensitive detection of candidate regions containing potential breakpoint, relaxed criteria are adopted to call breakpoints from chimeric/partial alignments, as well as from entirely aligned discordant pairs (p4 and p5). Subsequently, to reach high accuracy, *de novo* assembly is performed on a candidate region by using the read pairs anchored in this region. Lastly, *bona fide* breakpoints relative to the genome are identified from the assembled sequences. A fusion candidate is called if it attracts a sufficient number of supporting reads. While the mapping and assembly steps adopt the state-of-the-art algorithms, the breakpoint searching and fusion calling steps are novel (Materials and methods).

In order to detect fusion transcripts from PE RNA-seq data, we need to identify the fusion point from the sequencing read alignments. Discordant mapping of mate pairs, which include chimeric as well as partial alignments of an individual read, are reported by GSNAP [11] or STAR [12]. To guarantee high sensitivity, TRUP collects all candidate regions containing potential breakpoints suggested by those abnormal alignments. Additionally, for each candidate region, *de novo* assembly is performed using de Bruijn graphs (‘Velvet’) [13] and a modified version of Velvet (Oases) that employs additional filters to afford optimized merging of multiple assemblies, specifically of transcriptome sequencing data [14], with the aim to construct possible contigs from each region by leveraging dependency among reads. After sensitive split-read mapping and specific *de novo* assembly, fusion candidates are filtered and ranked based on repeat content and number of reads supporting the fusion points (Figure 1; Materials and Methods).

In order to evaluate the performance TRUP, we initially applied a preliminary version of TRUP (v1.0) to the well-characterized lung cancer cell-lines H3122 and H2228, which are known to harbor different variants of the *EML4-ALK* fusion gene [15], as well as to five lung adenocarcinoma tumor specimens that had been found positive for *ALK* rearrangements by FISH. On average, 50 million PE reads were uniquely mapped to the human genome (Additional file 1). We considered as high confidence candidates those chimeric transcripts that matched the following requirements: inter- or intra-chromosomal rearrangements; at least five independent reads supporting the breakpoint (either reads that span or read-pairs that encompass the fusion-point, referred as spanning reads and encompassing reads, respectively); and a non-repetitive sequence across the fusion-point (unless the chimeric transcript was also covered by encompassing reads). We found that below 5x most of the candidates called were artifacts of the pipeline or barely expressed chimeric transcripts difficult to validate by RT-PCR. In the seven samples analyzed, 20 chimeric transcripts matched the above-mentioned requirements. Out of these 20, 17 (85%) were validated by RT-PCR and Sanger sequencing across the fusion-point, or by FISH in the case of *EML4*-*ALK* (Table 1). These results were used to build an improved version of TRUP (v2.0), which not only recovered all the above-mentioned validated candidates but also identified 28 additional high-confident ones (Additional file 2). For all subsequent analyses version 2.0 was used.

**Table 1:** ***EML4-ALK*** **co-occurring fusion genes and chimeric transcripts detected with TRUP 1.0**

**PatID**	**HistoID**	**Chimeric_transcript**	**Sp**	**Enc**	**Total**	**Type-I**	**Type-II**	**Domains**	**Validated**
**H2228**	Cell-line	**EML4-ALK_v3**	**8 (8 + 8)**	**13**	**21**	**Intra**	**IF**	**Protein_kinase**	**.**
		SND1-CFTR	6 (6 + 4)	5	11	Intra	IF	Snase/ABC_tran	RT-PCR
		DCBLD2-STXBP5L	7 (7 + 2)	2	9	Intra	IF	CUB/LCCL/F5_F8_type_C	RT-PCR
**H3122**	Cell-line	SOS1-ADCY3	16 (16 + 15)	16	32	Intra	IF	RhoGEF/Guanylate_cyc	RT-PCR
		**EML4-ALK_v1**	**22 (22 + 7)**	**9**	**31**	**Intra**	**IF**	**Protein_kinase**	**.**
**S00006**	AD	**EML4-ALK_v2**	**15 (15 + 4)**	**5**	**20**	**Intra**	**IF**	**Protein_kinase**	**FISH**
**S00054**	AD	**EML4-ALK_v1**	**24 (24 + 16)**	**30**	**54**	**Intra**	**IF**	**Protein_kinase**	**FISH**
		PIGF-CHMP3	13 (13 + 0)	4	17	Intra	IF	Snf7	RT-PCR
		SNAP29-CELSR1	8 (8 + 2)	6	14	Intra	IF	EGF/LamininG2/LamininEGF/HRM/GPS/7tm2	RT-PCR
		APOBEC3F-SBF1	4 (4 + 0)	1	5			Not validated	
**S01122**	AD	**EML4-ALK_v1**	**29 (29 + 8)**	**12**	**41**	**Intra**	**IF**	**Protein_kinase**	**FISH**
		BMI1-ABI1	14 (14 + 4)	7	21	Intra	OF	.	RT-PCR
		MYO10-GPC5	12 (12 + 0)	0	12	Inter	IF	Myosin_head/IQ/PH	RT-PCR
		ARHGEF7-ZDHHC11	8 (8 + 2)	0	8	Inter	IF	CH/SH3/RhoGEF	RT-PCR
**S01124**	AD	**EML4-ALK_v1**	**0**	**4**	**4**	**Intra**	**IF**	**Protein_kinase**	**FISH**
		TAF4-LSM14B	12 (12 + 1)	2	14	Intra	IF	FDF	RT-PCR
**S01320**	AD	**EML4-ALK_v1**	**21 (21 + 7)**	**6**	**27**	**Intra**	**IF**	**Protein_kinase**	**FISH**
		NUP85-GPC3	12 (12 + 2)	0	12	Inter	IF	Nucleopor_Nup85	RT-PCR
		NR2C1-PTPRB	15 (15 + 4)	4	19			Not validated	
		KRIT-MAGI2	2 (2 + 3)	3	5			Not validated	

Table summarizing the chimeric transcripts detected in two lung cancer cell-lines and five lung adenocarcinoma (AD) *EML4-ALK*-positive tumors. Information about the EML4-ALK variant detected is indicated (v1, v2, v3). The number of spanning (Sp) and encompassing (Enc) reads is given, as well as additional information of the chimeric transcripts: intra-chromosomal (Intra), inter-chromosomal (Inter), in-frame (IF), out-of-frame (OF).

In order to determine the robustness and accuracy of TRUP we applied our pipeline to a published PE RNA-seq dataset of small cell lung cancer [16]. We applied TRUP to the cell-line data, where experimental validation of candidates was possible. We were able to identify two novel fusion transcripts affecting histone modifiers: one predicted to inactivate the histone acetyl-transferase CREBBP in the cell line, N417, and the other one leading to the inactivation of the TAF6-like RNA polymerase II p300/CBP-associated factor (PCAF)-associated factor (TAF6L) in the cell line, H187 (Additional file 3). These results are in agreement with previous studies in which alterations of histone modifiers by rearrangements were reported [17,18], and support the important role that these genes might have in the development and maintenance of small cell lung cancer.

### Detection of secondary rearrangements in *EML4-ALK* positive cases

**Figure 2:**
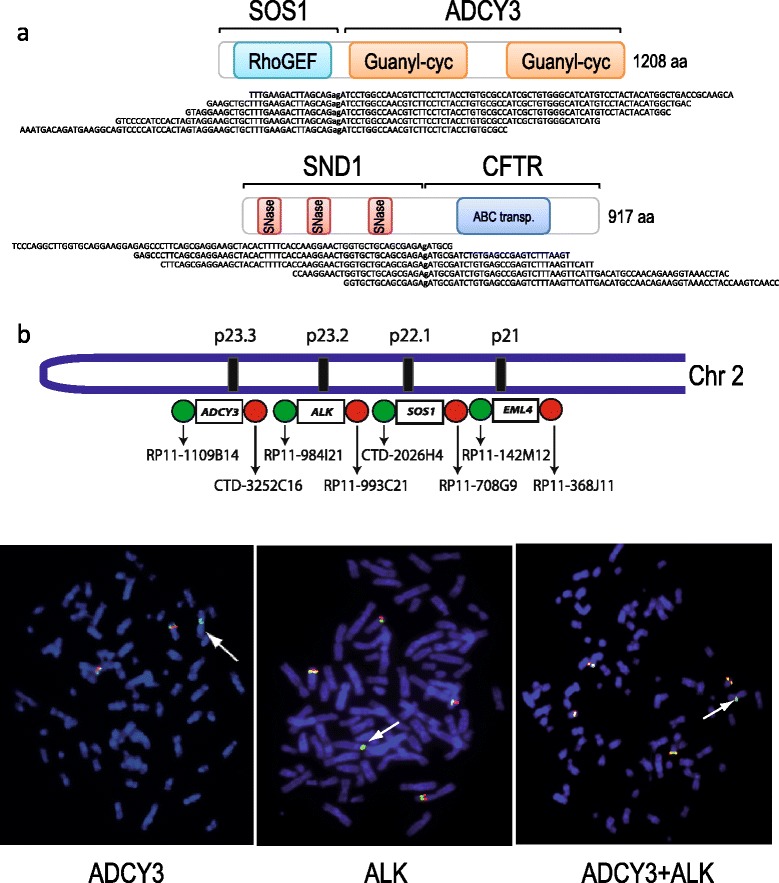
**Genomic complexity of** ***EML4-ALK*** **co-occurring fusion genes. (a)** Detection by transcriptome sequencing of SOS1-ADCY3 and SND1-CFTR in the H3122 and H2228 *EML4-ALK*-positive cell-lines, respectively. Schematic representation of the fusion transcripts and some of the transcriptome sequencing reads spanning the fusion point. **(b)** Top: schematic representation of part of chromosome 2 illustrating the location of *ADCY3*, *ALK*, *SOS1*, and *EML4* as well as the relative location of the FISH probes. Bottom, from left to right: *ADCY3* break-apart assay, *ALK* break-apart assay, and simultaneous application of *ADCY3* and *ALK* break-apart assays on metaphase chromosomes of H3122 cells.

Paired-end RNA-seq analysis of the *EML4-ALK* positive lung cancer cell-lines H3122 and H2228 revealed that in both cases *EML4-ALK* co-occurred with secondary in-frame chimeric transcripts: SOS1-ADCY3 in the case of H3122, and SND1-CFTR and DCBLD2-STXBP5L in the case of H2228 (Table 1; Figure 2a). We noticed that the genes involved in *EML4-ALK* and *SOS1-ADCY3* were located in the same region of chromosome 2 (Figure 2b, upper panel). In fact, the arrangement of these two genes in the genome suggested that *SOS1*-*ADCY3* might be generated by the same genomic event that had caused the *EML4*-*ALK* fusion. In order to test this hypothesis we first performed a break-apart FISH assay (ba-FISH) for both *SOS1* and *ADCY3* genes and a fusion assay for *SOS1-ADCY3* on H3122 interphase chromosomes, to test whether the alteration happened at the genomic level (Additional file 4). We then performed ba-FISH for both *ALK* and *ADCY3* separately, on metaphase chromosomes of the same cell line (Figure 2b, lower panel): in the case of *ADCY3* ba-FISH we found one aberrant single green signal with loss of the correspondent red signal. The same pattern was observed when performing the assay for *ALK*. We therefore reasoned that if both rearrangements were linked, when performing both assays together we should see the same pattern as observed separately (that is, one single green signal), since the two green signals would overlap and therefore be indistinguishable (Additional file 5, arrow A). On the contrary, if the two rearrangements occurred on different alleles, we should be able to distinguish two separate single green signals, one from the assay testing *ALK* and one for the assay assessing *ADCY3* (Additional file 5, arrow B). The combined *ALK*-*ADCY3* assay only generated one single green signal suggesting that the two rearrangements were likely to be physically linked (Figure 2b, lower panel). In addition to these two cell-lines, we validated at least one secondary in-frame chimeric transcript in four additional *EML4-ALK* positive primary tumors: SNAP29-CELSR1 and PIGF-CHMP3 in sample S00054; MYO10-GPC5 and ARHGEF7-ZDHHC11 in S01122; TAF4-LSM14B in S01124; and NUP85-GPC3 in S01320 (Table 1).

Although fusion genes are not necessarily expected to be accompanied by changes in gene copy number (for instance, balanced translocations are copy neutral alterations), for many of these samples, for which copy number data were available, we observed well-defined breakpoints suggesting these events happen at the genomic level and not as a consequence of trans-splicing [19] (Additional file 6). These data suggest that the recurrent fusions in lung cancer might be reciprocal and balanced rather than merely accompanied by broad destruction of otherwise non-oncogenic chromosomal DNA.

### Comparison of TRUP to alternative fusion detection tools

**Figure 3:**
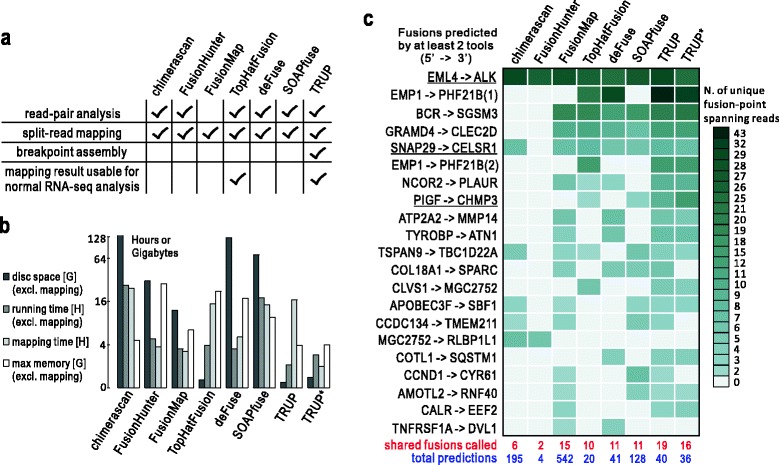
**Comparison between TRUP and other publically available fusion detection tools. (a)** Feature comparison: TRUP adopts breakpoint assembly after a sensitive detection of potential fusion points. Note that only TRUP and TopHat-Fusion are integrated into regular RNA-seq analysis pipelines, that is, the mapping results are shared for fusion detection and regular RNA-seq analysis. Alternative tools adopt various split-read mapping strategy specifically for fusion detection, generating customized mapping results, which could not be easily re-used for other purposes. **(b)** Computing resources consumed by TRUP and other tools for processing the data of sample S00054: resources used in the step of mapping are isolated for each tool to indicate the cost only for fusion detection and further processing (excl.: excluding). TRUP* indicates that TRUP is run with STAR as the mapper instead of GSNAP. **(c)** A heatmap showing the number of non-redundant reads spanning fusion-points for candidates predicted by at least two tools in sample S00054 (referred to as ‘shared fusions’ which is used as a gold set for evaluation). TRUP* indicates that TRUP is run with STAR as the mapper instead of GSNAP. Underlined fusions are experimentally validated.

In order to evaluate the performance of TRUP we applied eight additional fusion detection tools to the data of sample S00054: chimerascan [4], FusionHunter [20], FusionMap [9], TopHat-Fusion [8], deFuse [7], SOAPfuse [21], FusionSeq [6], and BreakFusion [10]. For two tools (FusionSeq and BreakFusion) evaluation could not be carried out because of computational limitations. Details about parameter settings are provided in Additional file 7. Despite the fact that most tools use both read-pair analysis and split-read mapping for the detection of fusion transcripts, they vary widely in terms of resources required and computing time (Figure 3a and b). Although TRUP additionally capitalizes on regional assembly of potential fusion-points, its overall performance in terms of disc space, memory size, and running time is superior to the others (Figure 3b). Also, even though the sensitive split mapping via GSNAP takes more time in the case of TRUP, the re-usability of the mapped data will eventually save time when regular RNA-seq analyses are performed. Alternatively, STAR can be used, as this mapper dramatically decreases the mapping time (Figure 3b) although it is a little less sensitive than GSNAP (Figure 3c). Notably, only the mapping results generated by TRUP and TopHat-Fusion are reusable for regular RNA-seq analysis, whereas other tools perform customized split-read mapping specifically for fusion detection (Figure 3a).

By using relaxed criteria requiring at least two supporting reads with a minimum of one read spanning the fusion-point, the seven tools reported from four to 542 fusion transcripts in sample S00054 (Figure 3c). Since the full set of true-positive predictions was not available we took the set of predictions shared across tools as gold set. There were in total 21 fusion transcripts detected by at least two independent tools, including three experimentally validated ones. Despite the low stringency settings, most alternative tools were only able to detect the *EML4-ALK* fusion but not the additional two experimentally validated chimeric transcripts. Three tools showed a high number of unique predictions suggesting a high number of false-positives. In case of FusionMap this might be due to the fact that this tool primarily relies on a single method (split-read mapping) not offering the use of a complementary approach for eliminating false-positives. By contrast, TRUP combines sensitive split-read alignment and discordant read-pair analysis with regional breakpoint assembly, thus achieving a better balance between sensitivity and total number of predictions.

Due to the dependence on partial and chimeric alignments for the selection of potential breakpoints, GSNAP becomes the first choice owing to its ability in sensitive split-read mapping although with a relatively slow speed. Since runtime might be a limiting factor in large-scale projects we optionally provide the extremely fast mapper STAR as an alternative to GSNAP. When using STAR to process sample S00054, the performance of TRUP remains high (Figure 3c) with a much-reduced running time (Figure 3b). Sixteen out of 21 fusions in the gold set were found by TRUP with STAR (36 fusions predicted in total), showing a slightly lower recall and comparable precision as compared to GSNAP (19 found, 40 predicted). Nevertheless, such a recall is still higher than that of other fusion prediction tools.

**Figure 4:**
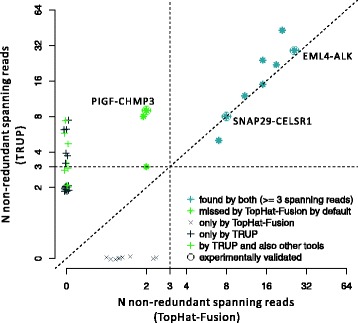
**Comparison of TopHat-Fusion with TRUP analysis tools.** The fusion candidates found by TRUP and TopHat-Fusion on sample S00054, plotted by the number of non-redundant spanning reads reported by the two tools. The horizontal and vertical dashed lines indicate the threshold of three spanning reads for calling a chimeric transcript. The diagonal dashed line is plotted to show that TRUP usually reports more reads spanning a fusion point. The axes are in log2 scale. The unique calls to each algorithm are jittered to avoid over-plotting.

We compared TopHat-Fusion and TRUP in more detail since they showed the best performance (the harmonic mean of recall and precision for TRUP is 0.62 and for TopHat-Fusion is 0.49, highest among all the tools) with a total of 20 and 40 predicted fusion events, respectively (Additional files 8 and 9). For candidates with very low coverage, disagreements between the two tools were observed, indicating higher uncertainty for calling fusion transcripts with low expression. After manually checking the calls unique to TRUP, we only found a single candidate that might be considered as a false positive. This candidate exhibited a breakpoint located in a repetitive region and also showed a low spanning score, which summarizes the confidence of supporting evidence of the spanning reads (Materials and methods). In order to avoid using suboptimal parameter setting for TopHat-Fusion, we alternatively used the default settings and adjusted the TRUP parameters accordingly. We therefore increased the threshold for fusion calling as follows: presence of at least three reads spanning the fusion point and two encompassing mate pairs. TopHat-Fusion now detected eight fusion events all of which were included in the 25 candidates found by TRUP (Figure 4). Both tools successfully recovered the *EML4-ALK* fusion as well as one of the secondary fusions, *SNAP29-CELSR1*. However, the fusion event *PIGF-CHMP3* was only reported by TRUP. TopHat-Fusion failed to call this true positive because the number of spanning reads was limited to two. By contrast, TRUP detected nine fusion-spanning reads. We found that TRUP usually reports more non-redundant spanning reads than TopHat-Fusion, indicating a higher sensitivity in identifying reads showing chimeric patterns. Judging from the results of the analysis of sample H3122, TRUP performs well even on paired-end data with short insert sizes (here 70 bp) where mate pairs overlap.

Taken together, TRUP achieves a better balance among recall, precision, and computational efficiency for the detection of fusion events from RNA-seq data compared to the alternative tools tested here. Similar to TopHat-Fusion, TRUP can be used in a standard RNA-seq pipeline thus diminishing the impact of the sensitive but slower spliced-mapping procedures of GSNAP. Faster aligners, such as STAR, can also be used in TRUP if running time is the major concern.

### Recurrent inactivating *RASSF8* rearrangements in cancer

**Figure 5:**
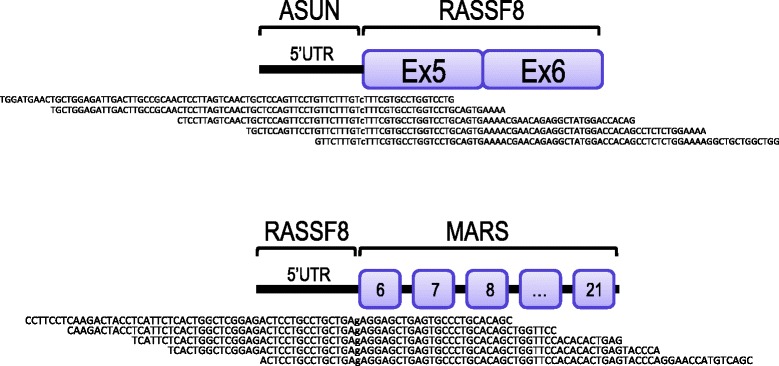
**Recurrent inactivating rearrangements of** ***RASSF8*** **in cancer.** Identification of ASUN-RASSF8 in a lung adenocarcinoma tumor (upper panel) and RASSF8-MARS in the KPD osteosarcoma cell-line (lower panel). Schematic representation of the fusion transcripts and some of the transcriptome sequencing reads spanning the fusion point.

We next applied the method to 17 additional primary lung adenocarcinoma specimens (Additional file 10). Of the 17 samples analyzed, one carried an inactivating chimeric RASSF8 transcript (Figure 5, upper panel; Additional file 11a). The sample was negative for *EGFR* or *KRAS* mutations and belonged to the adenocarcinoma of a current smoker (Additional file 10). *RASSF8* is one of the four N-terminal RASSF proteins (RASSF7-10) that belong to the Ras-association-domain-containing family of proteins, which also include the classical RASSF proteins (RASSF1-6) that are known to act as tumor suppressors and are frequently epigenetically silenced in tumors [22]. In order to further investigate the role of RASSF8 in lung adenocarcinoma, we silenced *RASSF8* expression in the lung cancer cell line, H1395, which expresses wild-type *RASSF8*. In comparison to the EGFP transfected cells, silencing of *RASSF8* led to a significant increase of cell proliferation of more than 60% (*P* <0.0001) (Additional file 11b). RASSF8 was not completely silenced, as detected by western blotting (Additional file 11c), suggesting that low doses rather than complete loss of the RASSF8 protein is sufficient to induce cell proliferation. Furthermore, we identified an inactivating rearrangement of *RASSF8* in the osteosarcoma cell line, KPD (Figure 5, lower panel; Additional file 11a). The breakpoint of this translocation event was also detectable when analyzing the copy number data (Additional file 12) suggesting that the rearrangement happened at the genomic level.

RASSF8 has been proposed as a new tumor suppressor in lung cancer. However, genetic data to support this notion have so far been missing [22-24]. Our data thus provide further support for a role of RASSF8 as a tumor suppressor and suggest that genomic translocations might be a relevant mechanism for the genetic inactivation of *RASSF8*.

## Conclusions

Taken together, TRUP is a new tool for the identification of chimeric transcripts using PE RNA-seq data, which shows a balance between sensitivity, specificity, and computational efficiency for prediction of fusion events. TRUP afforded the identification of new fusion events in the context of *EML4-ALK*, which were genomically linked in one case, suggesting that in some cases *EML4-ALK* occurs as a balanced translocation. We furthermore detected inactivating rearrangements affecting *RASSF8*, supporting its role as a tumor suppressor gene in cancer.

## Materials and methods

### Sample preparation, DNA and RNA extraction, and illumina sequencing

Sample preparation and DNA and RNA extraction were performed as previously described [18]. RNAseq was performed on cDNA libraries prepared from PolyA+ RNA extracted from tumor cells using the Illumina TruSeq protocol for mRNA. The final libraries were sequenced with a paired-end 2 × 100 bp protocol aiming at 8.5 Gb per sample, resulting in a 30× mean coverage of the annotated transcriptome. All the sequencing was carried out on an Illumina HiSeq 2000 sequencing instrument (Illumina).

### Analysis of chromosomal gene copy number (SNP 6.0)

Hybridization of the Affymetrix SNP 6.0 arrays was carried out according to the manufacturers’ instructions and analyzed using a previously described method [18].

### TRUP pipeline

#### Development of TRUP (version 1.0 to 2.0)

The differences between 1.0 and 2.0 TRUP are two-fold: (1) the mapping tool: from TopHat to GSNAP; and (2) whereas TRUP 1.0 feeds the total (pooled) abnormal reads into assembly, TRUP 2.0 assembles the reads surrounding each potential breakpoint detected from GSNAP alignments. In the early development stage TRUP 1.0 was tested on cell line data. The validation of the predictions was used to guide us to improve TRUP to 2.0. Below only the strategy of TRUP 2.0 is described as the old version 1.0 is deprecated.

#### Sensitive split-read and read pair mapping against the genome

TRUP maps PE RNA-seq reads onto the Hg19 reference genome using GSNAP, a hash-table based spliced aligner [11]. It breaks a read into short seeds to localize the alignment, followed by iterative extension of candidate regions and merging of initial seeds to the exact spliced alignment. It has been shown that GSNAP predicts splicing alignments with high sensitivity among many available RNA-seq mappers [25], partly due to its unbiased evaluation of both un-spliced and spliced mapping of the same read [26]. Besides the ability to map splicing junction reads, GSNAP (version 2013-09-30 is used in this study) can report chimeric and partial alignments of an individual read, which are usually un-mappable by other mappers. A read is chimeric if it spans two different chromosomes/strands or a longer distance than the maximum allowed intron length. Partially aligned reads contain unmappable sequences at one of its ends that are clipped. These reads are the source for the identification of possible fusion junctions. In addition to GSNAP, a recent mapper, STAR, can also be used for the read alignment in TRUP (version 2.4.0 is tested).

#### Relaxed detection of candidate regions containing breakpoints

TRUP searches for chimeric and partial alignments indicating reads spanning possible fusion points. The information about discordant pairs is also incorporated. A read pair is discordant if the two ends are mapped to two different chromosomes, different strands or locations with a distance longer than the maximum allowed intron length. Chimeric reads are split and aligned as discordant pairs. A potential breakpoint is called from a read *X* with length *l* if it satisfies one of the following criteria (strength of evidence from high to low, *t*_*N*_ are user defined thresholds whose default is applied): (1) *X* shows chimeric mapping; (2) *X* is partially aligned with a discordant mate and the unmapped part should be at least (*kmer* < =1/5 *l* < =3/2 *kmer*) bp in size with no undecided nucleotides. *kmer* is set in the genome database for GSNAP (16 bp or less). Within the nearby ±200 bp region of *X*, TRUP requires the existence of at least t_1_ (default: 2) other supporting read alignments with discordant mates consistent with *X*. (iii) For partially aligned *X* with a shorter unmapped segment having a discordant mate, TRUP requires at least t_2_ (default: 3) other discordant read alignments consistent with *X*. (iv) For a group of t_3_ (default: 4) entirely aligned discordant read pairs that are all consistent with a fusion point, the potential breakpoint will be set to the locations where the reads’ 3′ ends extend farthest. If STAR is used as mapper, the chimeric junctions for each read produced by STAR are incorporated into the breakpoint calls, in addition to the ones identified by scanning the mapping results.

TRUP groups the breakpoint calls if they are within 200 bp in distance after removing calls from repetitive region. The call with the strongest evidence will be used as the group representative. A candidate region is defined as ±250 bp centered on each breakpoint call.

#### De novo assembly of read pairs anchored in candidate regions

TRUP extracts the abnormal read pairs (discordant pairs, singletons, and reads showing partial or chimeric alignments) anchored in a candidate region and feeds them into a regional assembly by using Velvet [13] and Oases [14]. Oases is a Bruijn graph-based assembler that receives a preliminary assembly produced by Velvet as input. Oases is sufficiently sensitive and accurate to assemble possible alternative isoforms throughout a wide spectrum of expression levels [14].

#### Filtration and prioritization of fusion candidates

The assembled junction sequences are aligned back to the reference genome using BLAT [27]. After removing the non-unique segment hits (that is, mapping to multiple regions), TRUP concatenates the remaining partial alignments into longer ones, sometimes resulting in alternative mappings of the assembled sequence. The best and second best concatenated alignments (with aggregated score q_1_ and q_2_) for each assembled sequence with length q_0_ are used to calculate an alignment score (Q) as adopted by BreakFusion [10], where $Q=e\frac{q_1-{q}_0}{10}-e\frac{q_2-{q}_0}{10}$. TRUP then queries for those candidate sequences with their best paths exhibiting partial alignments, with minimal overlap (15 nt), to two different genes representing putative fusion junctions. TRUP flags the candidates whose fusion points are located in repetitive regions or UCSC Self-Chain annotated regions, both of which can indicate possible misalignments or incorrect assemblies due to sequence similarities.

Read pairs which are improperly aligned (including both assembly-derived and initially unmapped read pairs) can be either a direct support for a putative fusion junction if one end spans the fusion point with at least 13 nt aligned on both sides, or an indirect support that encompasses the fusion point. The junctions supported by at least two non-redundant supporting reads are reported. TRUP ranks the fusion candidates based on the following parameters applied in order: (1) the Spanning Score $SS=N-{\displaystyle \sum_{i-1}^N\frac{\left|{L}_i-{R}_i\right|}{L_i+{R}_i}}$ that takes into account both the number of independent spanning reads *N* and the mapping balance of each spanning read i on the left and right side of the fusion point (with mapping distance *L*_*i*_ and *R*_*i*_, respectively); (ii) the number of independent encompassing read pairs; (iii) the alignment score of junction sequence *Q*. An additional filtration step was used to filter out those predictions with low (less than 5) total supporting reads (non-redundant), with low spanning score (less than 1) and/or those with both breakpoints residing in self-chain region or repetitive region. Isoform junctions for the same gene fusion were merged.

TRUP also has several other modules for RNA-seq analysis in general, such as quality assessment, gene/transcript expression quantification, and differential expression analysis. TRUP is available at [28].

### *ADCY3*, *SOS1*, *ALK*, and *EML4* FISH break apart assays

A dual-color break-apart fluorescence in-situ hybridization (FISH) assay was developed to assess for *ADCY3*, *SOS1*, *ALK*, and *EML4* (chromosome 2) rearrangements on the chromosomal level as described earlier [29]. All centromeric BAC clones were labeled red using biotin and all telomeric BAC clones were labeled green using digoxigenin. In brief, for the *ADCY3* break-apart assay, we used the BAC clone CTD-3252C16 for centromeric labeling with biotin (eventually producing a red signal) and RP11-1109B14 for telomeric labeling with digoxigenin (eventually producing a green signal). Similarly, for the *SOS1* break-apart assay, we used BAC clone RP11-708G9 for centromeric labeling and CTD-2026H4 for telomeric labeling, for the *ALK* break-apart assay RP11-993C21 was used as the centromeric probe and RP11-984I21 was used as the telomeric probe, and for the *EML4* break-apart assay, RP11-368 J11 was used as the centromeric labeled probe and RP11-142 M12 was used as the telomeric labeled probe. Metaphase spreads were prepared as previously described [29]. FISH on the metaphase spreads was performed by pre-treating the slides with 2x SSC solution and digesting it with Digest-All III (dilution 1:2). FISH probes were added to the metaphase spreads and co-denatured at 85°C for 4 min. and hybridized overnight at 37°C. Post-hybridization, slides were washed with 0.5x SSC and streptavidin-Alexa-594 conjugates (dilution 1:200) and anti-digoxigenin-FITC (dilution 1:200) were added to the slides. Counterstaining was performed using 4′,6-Diamidin-2′ phenylindoldihydrochlorid (DAPI) and mounted. All slides were analyzed under a 63x oil immersion objective using a fluorescence microscope (Zeiss, Jena, Germany) and images were captured using the Metafer 4 software (Metasystems, Altlussheim, Germany). Assessment of the experiments was done independently by two evaluators (RM and SP). Gene rearrangements were defined as follows: a loss of a signal, resulting in either a single red or single green signal for at least one allele is referred to as a rearrangement through deletion, or a wild-type allele displays a juxtaposed red and green signal (mostly forming a yellow signal).

### *RASSF8* knockdown

A total of 60,000 cells per well were seeded in 2.5 mL culture media in six well plates 1 day before the transfection. Triplicates were made. For the transfection 1.5 mL of Opti-MEM and 15 μL of Lipofectamine RNAiMax (Life Technologies), were mixed and incubated for 5 min at room temperature. After 5 min 400 ng of esiRNA were incubated for 20 min. Then 500 μL of the mix were added dropwise to each well. After 6 days cells were counted. Additionally, the triplicates were pulled, centrifuged 5 min, 4°C, 13,500 rpm, washed with PBS and resuspended with lysis buffer (1 mL of 10x lysis buffer (Cell Signaling), one tablet of Protease Inhibitor (Roche), 200 μL of phosphatase inhibitor cocktail III (Merck), 5 μL of 200 mM PMSF (Carl Roth), filled up to 10 mL). After 10 min incubation on ice, samples were centrifuged 10 min, 4°C, 13,500 rpm, and supernatants were transferred to new tubes. Protein determination was assessed with BCA Kit (Pierce). Western blot was performed according with standard procedures. The antibodies used were: RASSF8 (4B1) mouse monoclonal (Santa Cruz, 1:250), goat anti mouse HRP (Millipore, 1:3,000), and ß-actin HRP conjugated (Santa Cruz, 1:3,000).

### Accession codes

Transcriptome sequencing data and affymetrix 6.0 (copy number) data have been deposited at the European Genome-phenome Archive under the accession code EGAS00001000659.

## Additional files

Additional file 1:
**Overview RNA-seq data generated.**
Additional file 2:
**Fusions predicted by TRUP 2.0 in 6** ***EML4-ALK*** **-positive samples (S00054 is shown in Additional file** 8 **).**
Additional file 3:
**Overview chimeric transcripts detected in SCLC cell-lines involving histone modifiers.**
Additional file 4:
**FISH patterns of the** ***ADCY3*** **and** ***SOS1*** **break-apart, and** ***SOS1-ADCY3*** **fusion assay, on H3122.**
Additional file 5:
**Explanatory schema of FISH experiment shown in Figure** 2 **b.**
Additional file 6:
**Fusion breakpoints inferred from copy number data.**
Additional file 7:
**Parameter settings for different fusion detection tools on sample S00054.**
Additional file 8:
**TRUP predictions in sample S00054.**
Additional file 9:
**TopHat-Fusion predictions in sample S00054.**
Additional file 10:
**Overview chimeric transcripts detected and validated in lung adenocarcinoma tumors, annotated for** ***KRAS*** **and** ***EGFR*** **mutations.**
Additional file 11:
**Inactivation of** ***RASSF8*** **in cancer.**
Additional file 12:
***RASSF8*** **breakpoint in KPD osteosarcoma cell line inferred from copy number data and RNA-seq data.**
