# Supplementary material for: Identification of novel fusion genes in lung cancer using breakpoint assembly of transcriptome sequencing data
Source: Genome Biol. 2015 Jan 5;16(1):7. doi: 10.1186/s13059-014-0558-0 (PMC4300615; doi:10.1186/s13059-014-0558-0)
Supplement: Additional file 1: — Overview RNA-seq data generated. [file 13059_2014_558_MOESM1_ESM.docx]

**Additional file 1. Overview RNA-seq data generated**
