# Supplementary material for: Identification of novel fusion genes in lung cancer using breakpoint assembly of transcriptome sequencing data
Source: Genome Biol. 2015 Jan 5;16(1):7. doi: 10.1186/s13059-014-0558-0 (PMC4300615; doi:10.1186/s13059-014-0558-0)
Supplement: Additional file 2: — Fusions predicted by TRUP 2.0 in 6 EML4-ALK -positive samples (S00054 is shown in Additional file 8 ). [file 13059_2014_558_MOESM2_ESM.docx]

**Additional file 2. Fusions predicted by TRUP 2.0 in 6 *EML4-ALK*-positive samples (S00054 is shown in Additional file 8).**
