# Supplementary material for: Identification of novel fusion genes in lung cancer using breakpoint assembly of transcriptome sequencing data
Source: Genome Biol. 2015 Jan 5;16(1):7. doi: 10.1186/s13059-014-0558-0 (PMC4300615; doi:10.1186/s13059-014-0558-0)
Supplement: Additional file 3: — Overview chimeric transcripts detected in SCLC cell-lines involving histone modifiers. [file 13059_2014_558_MOESM3_ESM.docx]

**Additional file 3. Overview chimeric transcripts detected in SCLC cell-lines involving histone modifiers.** Summary table and schematic representation of the fusion transcripts and the transcriptome sequencing reads spanning the fusion point.

Sp: spanning reads

Enc: encompassing reads

OF: out-of-frame
