# Supplementary material for: Identification of novel fusion genes in lung cancer using breakpoint assembly of transcriptome sequencing data
Source: Genome Biol. 2015 Jan 5;16(1):7. doi: 10.1186/s13059-014-0558-0 (PMC4300615; doi:10.1186/s13059-014-0558-0)
Supplement: Additional file 4: — FISH patterns of the ADCY3 and SOS1 break-apart, and SOS1-ADCY3 fusion assay, on H3122. [file 13059_2014_558_MOESM4_ESM.docx]

**Additional file 4. FISH patterns of the *ADCY3* and *SOS1* break-apart, and *ADCY3*-*SOS1* fusion assay, on H3122.** From left to right: *ADCY3* break-apart assay, *SOS1* break-apart assay, and *SOS1-ADCY3* fusion assay on H3122 cells. Arrows show aberrant signals
