# Supplementary material for: Identification of novel fusion genes in lung cancer using breakpoint assembly of transcriptome sequencing data
Source: Genome Biol. 2015 Jan 5;16(1):7. doi: 10.1186/s13059-014-0558-0 (PMC4300615; doi:10.1186/s13059-014-0558-0)
Supplement: Additional file 5: — Explanatory schema of FISH experiment shown in Figure 2 b. [file 13059_2014_558_MOESM5_ESM.docx]

**Additional file 5. Explanatory schema of FISH experiment shown in Figure 2b.** Top: arrangement of genes in chromosome two. *EML4* is located in the positive strand while *ADCY3*, *ALK*, and *SOS1* are found in the negative strand. FISH images: the FISH pattern shows that, as expected, the C-terminal part of *ADCY3* and *ALK* genes (labeled in green) is present while the N-terminal part (labeled in red) is lost in the allele undergoing the translocation. Arrow A: predicted *ALK*+*ADCY3* assay FISH pattern if both translocations (*EML4-ALK* and *SOS1-ADCY3*) happened in the same allele. Arrow B: predicted *ALK+ADCY3* assay FISH pattern if both translocations happened in different alleles.
