# Supplementary material for: Identification of novel fusion genes in lung cancer using breakpoint assembly of transcriptome sequencing data
Source: Genome Biol. 2015 Jan 5;16(1):7. doi: 10.1186/s13059-014-0558-0 (PMC4300615; doi:10.1186/s13059-014-0558-0)
Supplement: Additional file 7: — Parameter settings for different fusion detection tools on sample S00054. [file 13059_2014_558_MOESM7_ESM.docx]

**Additional file 7. Parameter settings for different fusion detection tools on sample S00054**

For **chimerascan (v 0.4.5)**, we set the following parameters:

**-v** **--processors** 8 **--isize-mean** 210 **--isize-stdev 35 --filter-unique-frags** 2

The command line execution is:

python chimerascan-0.4.5/bin/chimerascan_run.py -v -p 8 --isize-mean 210 --isize-stdev 35 --filter-unique-frags 2 chimericScan/ref/(reference files) S00054_1.fq S00054_2.fq output_dir/

For **FusionHunter (v. 1.4),** we set the following parameters:

In the configuration file, we set '**segment_size**' as 32 (approximately one third of the read length); '**CORE**' as 8, '**PAIRNUM**' as 2; '**MINSPAN**' as 1

The command line execution is:

Perl FusionHunter-v1.4-Linux_x86_64/bin/FusionHunter.pl FusionHunter.cfg (config file)

For **FusionMap (v. 2014-01-01 for linux),** we set the following parameters:

In the control file, we set '**ThreadNumber**' as 8; '**FusionReportCutoff**' as 2, '**MinimalFusionSpan**' as 230000

The command line execution is:

mono-2.10.9/bin/mono FusionMap_2014-01-01/bin/FusionMap.exe --semap references/ Human.hg19 Ensembl.R72 AllOptions.config >run.log

For **TopHat-Fusion’s tophat (v. 2.0.8b)** mapping step, we used the following flags:

**--allow-indels** **–p** 8 **--no-coverage-search** **--mate-inner-dist** 20 --mate-std-dev 35 **--max-intron-length** 23000 **--fusion-anchor-length** 13 **--fusion-ignore-chromosomes** chrM

The command line execution is:

tophat-2.0.8b.Linux_x86_64/tophat -o output_dir/ -p 8 --fusion-search --keep-fasta-order --bowtie1 --allow-indels --no-coverage-search --mate-inner-dist 24 --mate-std-dev 35 --max-intron-length 230000 --fusion-min-dist 230000 --fusion-anchor-length 13 --fusion-ignore-chromosomes chrM --transcriptome-index bowtie_index/hg19 S00054_1.fq.gz S00054_2.fq.gz

For **TopHat-Fusion (v. 2.0.8b)** **tophat-fusion-post** step, we used the following flags:

**--num-fusion-reads** 2 **--num-fusion-pairs** 1 **--num-fusion-both** 2 **--num-threads** 8

The command line execution is:

tophat-2.0.8b.Linux_x86_64/tophat-fusion-post --num-fusion-reads 2 --num-fusion-pairs 1 --num-fusion-both 2 --num-threads 8 bowtie_index/hg19

For **defuse (v. 0.6.1)**, we used the following parameters:

In the configure file, we set '**ucsc_genome_version**' as hg19, '**bowtie_threads**' as 8, '**split_min_anchor**' as 5, '**span_count_threshold**' as 2.

The command line execution is:

Perl defuse-0.6.1/scripts/defuse.pl -c config.file -1 S00054.fq -2 S00054.fq -o output_dir -p 8

For **SOAPfuse (v. 1.26)**, we used the following parameters:

In the config.txt (configuration) file, we set '**PA_all_process_of_align_software**' as 8, '**PA_s05_the_minimum_span_reads_for_candidate**' as 2, '**PA_s08_min_intrachr_distance**' as 230000.

The command line execution is:

perl SOAPfuse-v1.26/SOAPfuse-RUN.pl -c config.txt -fd data/ -l samplelist

For **TRUP (v. 2.0)**,

The command line execution:

Perl RTrace.pl --runlevel 1-4 --sampleName S00054 –seqType p --root analysis_dir/ --species hg19 --anno annotation_dir/ --readpool reads_dir/ --mapper gsnap/star –uniqueBase 295 –misPen 3 --threads 8 2>>run.log

It should be noted that we initially included BreakFusion [10] and FusionSeq [6] but abandoned them due to their computational limitations. Unfortunately, BreakFusion is the only tool that adopts regional assembly for fusion detection. However, the performance of BreakFusion seems to depend on the alignment tool chosen by the user. In our case, using mapping results of GSNAP led to a surprisingly long running time, suggesting that the current version of BreakFusion and GSNAP are incompatible. The reason to abandon FusionSeq was the generation of a huge amount (approaching1TB) of temporary files (similar observation is reported in [20]).
