# Supplementary material for: Identification of novel fusion genes in lung cancer using breakpoint assembly of transcriptome sequencing data
Source: Genome Biol. 2015 Jan 5;16(1):7. doi: 10.1186/s13059-014-0558-0 (PMC4300615; doi:10.1186/s13059-014-0558-0)
Supplement: Additional file 9: — TopHat-Fusion predictions in sample S00054. [file 13059_2014_558_MOESM9_ESM.docx]

**Additional file 9. TopHat-Fusion predictions in sample S00054**
