# Supplementary material for: Identification of novel fusion genes in lung cancer using breakpoint assembly of transcriptome sequencing data
Source: Genome Biol. 2015 Jan 5;16(1):7. doi: 10.1186/s13059-014-0558-0 (PMC4300615; doi:10.1186/s13059-014-0558-0)
Supplement: Additional file 10: — Overview chimeric transcripts detected and validated in lung adenocarcinoma tumors, annotated for KRAS and EGFR mutations. [file 13059_2014_558_MOESM10_ESM.docx]

**Additional file 10. Overview chimeric transcripts detected and validated in lung adenocarcinoma tumors, annotated for *KRAS* and *EGFR* mutations**

Sp: spanning reads

Enc: encompassing reads

OF: out-of-frame

IF: in-frame
