# Supplementary material for: Identification of novel fusion genes in lung cancer using breakpoint assembly of transcriptome sequencing data
Source: Genome Biol. 2015 Jan 5;16(1):7. doi: 10.1186/s13059-014-0558-0 (PMC4300615; doi:10.1186/s13059-014-0558-0)
Supplement: Additional file 11: — Inactivation of RASSF8 in cancer. [file 13059_2014_558_MOESM11_ESM.docx]

**Additional file 11. Inactivation of *RASSF8* in cancer.** (**a**) Overview of *RASSF8* inactivating chimeric transcripts detected in cancer samples. (**b**) Impact of 6-days knockdown of *RASSF8* on H1395 lung cancer cells proliferation (Materials and Methods). We use a two-tailed, paired-T test for statistical analysis. *P* value <0.001 is indicated by ***. (**c**) Impact of 6-days knockdown of *RASSF8* on RASSF8 protein expression in comparison to EGFP control.

**a**

**b**

**c**
