# Supplementary material for: Identification of novel fusion genes in lung cancer using breakpoint assembly of transcriptome sequencing data
Source: Genome Biol. 2015 Jan 5;16(1):7. doi: 10.1186/s13059-014-0558-0 (PMC4300615; doi:10.1186/s13059-014-0558-0)

**Additional file 12. *RASSF8* breakpoint in KPD osteosarcoma cell line inferred from copy number data and RNA-seq data.** Top: breakpoint close to *RASSF8* in KPD osteosarcoma cell line inferred from copy number data. Copy number segments (red, copy number gain) inferred from SNP 6.0 arrays. Bottom: the RNA-seq reads in the vicinity of the three identified *RASSF8-MARS* fusion points. The reads are colored based on the insert size. Read colors indicate one end mapping to *RASSF8* and the other one mapping to *MARS*.


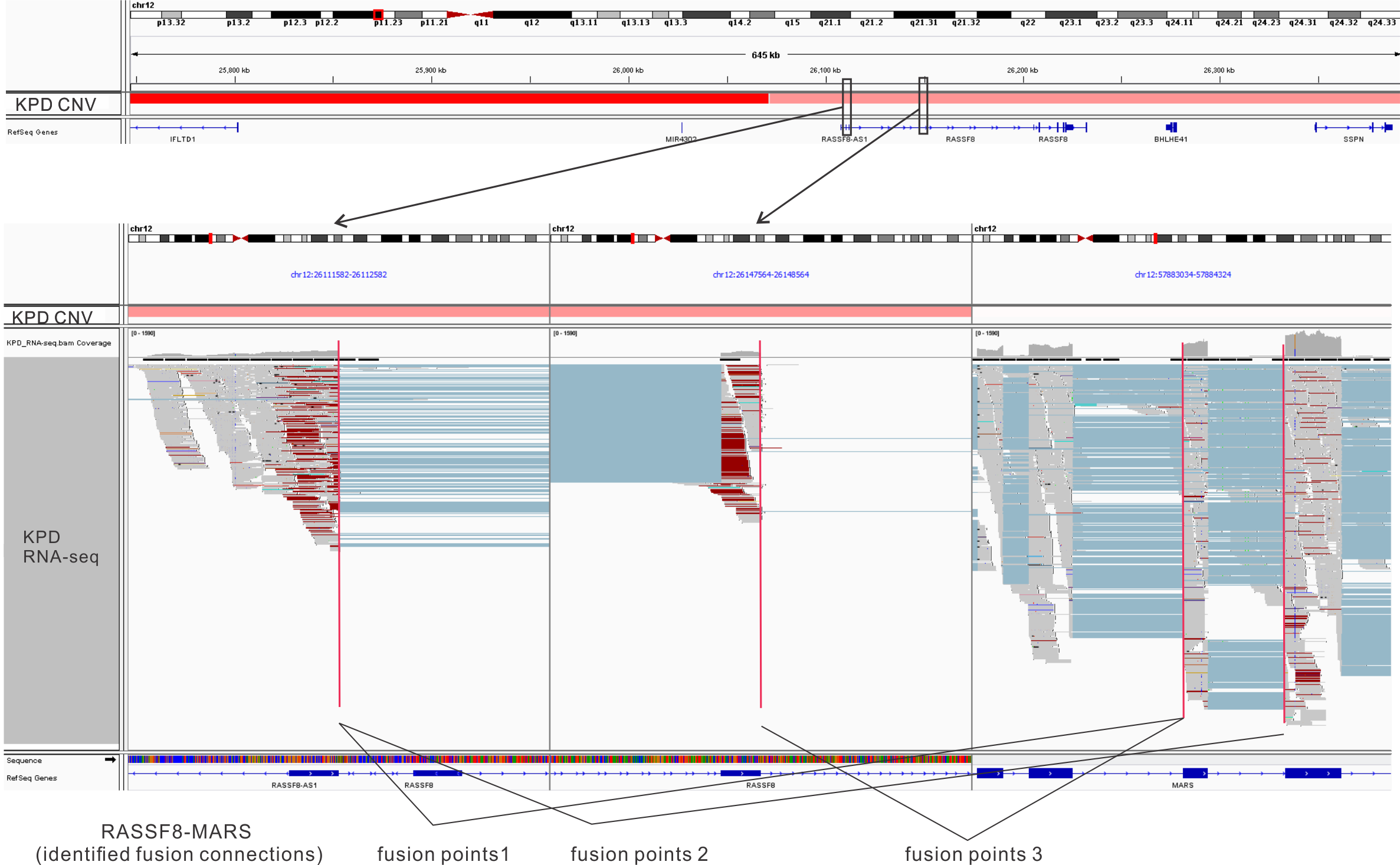

Supplement: Additional file 12: — RASSF8 breakpoint in KPD osteosarcoma cell line inferred from copy number data and RNA-seq data. [file 13059_2014_558_MOESM12_ESM.docx]
